# Communication Strategies to Promote Patient Engagement in Telemedicine: Systematic Review

**DOI:** 10.2196/85456

**Published:** 2026-01-21

**Authors:** Yangna Hu, Cindy Sing Bik Ngai, Rui Jiang

**Affiliations:** 1 Department of Language Science and Technology Faculty of Humanities Hong Kong Polytechnic University Hong Kong China (Hong Kong)

**Keywords:** communication strategies, patient engagement, telemedicine, health care services, provider-patient interactions

## Abstract

**Background:**

The rapid growth of telemedicine offers convenience, flexibility, and accessibility for patients to have health care services worldwide. To succeed in telemedicine, health care practitioners and telemedicine tools must engage patients through effective communication. However, a research gap exists in understanding the communication strategies used in telemedicine and how they effectively engage patients.

**Objective:**

This study aims to identify communication strategies influencing patient engagement in telemedicine with provider-patient interactions, as well as how included studies evaluate patient engagement through a systematic review.

**Methods:**

We searched the literature comprehensively using 6 databases, Web of Science, PubMed, Scopus, MEDLINE, CINAHL, and Embase, from inception to October 2025. We included empirical, English-language studies that examined communication strategies affecting patient engagement in telemedicine with provider-patient interactions. Studies lacking actual patients or provider-patient interactions in telemedicine were excluded. We used content analysis to identify texts that were related to Theme 1: the communication strategies affecting patient engagement, and Theme 2: evaluation of patient engagement. Coded texts were analyzed to develop subthemes and themes of identified communication strategies. Methods for evaluating patient engagement were summarized. A narrative synthesis was conducted because of heterogeneity across study design and outcomes. We used the Mixed Methods Appraisal Tool to assess the quality of research included in this study.

**Results:**

This study systematically reviewed 34 peer-reviewed articles, revealing 3 overarching themes of effective communication strategies that enhance patient engagement: interpersonal communication strategies, with 6 subthemes (building relationships, supportive attitude, interactive dialogic loop, nonverbal communication, professionalism and accuracy, and tailored communication); team-level communication strategies, with 3 subthemes (training and preparation, teamwork and care coordination, and cultural and linguistic sensitivity); and system-level communication strategies, with 3 subthemes (usefulness of information, ease of use, and data privacy and security). We also found that included studies predominantly used qualitative research methods, such as semistructured interviews and focus groups, to collect patient engagement data.

**Conclusions:**

This review provides an innovative synthesis of communication strategies that promote patient engagement in telemedicine by integrating interpersonal (micro), team (meso), and system-level (macro) perspectives. Unlike previous reviews that focused on single aspects or levels of communication, this study offers a holistic framework that advances theoretical understanding of how multilevel communication strategies collectively shape patient engagement. Practically, the findings offer actionable guidance for health care professionals, telemedicine developers, and policymakers seeking to enhance the quality and sustainability of telemedicine services. In real-world settings, the identified strategies can inform professional training, platform design, and policy development to support patient-centered digital care. This review is the first to systematically bring together communication strategies for patient engagement in telemedicine across all 3 levels. Future research should build on this framework by developing and validating quantitative measures of patient engagement and examining the relationships between communication strategies and telemedicine outcomes.

## Introduction

### Background

Digitally accessed health care has accelerated globally, prompted not only by the advancement of communication technologies but also by the increasing demand for accessible and efficient care delivery [1,2]. Consequently, the global use of telemedicine services has grown substantially, with an estimated compound annual growth rate of around 24% between 2022 and 2032 [3]. Telemedicine involves the delivery of health care services via the use of ICTs to engage health care providers (HCPs), patients, and caregivers, and improve health care outcomes [4-6]. It offers convenience and flexibility for both patients and providers and reduces medical service costs and patient wait times [7-11]. Furthermore, it significantly contributes to medical resource allocation, improving patient access and helping health care departments in low-resource settings address resource shortages [6,12-14]. A study analyzed telemedicine consultations in a university-based outpatient telemedicine program and found that the average savings per consultation were 278 miles, 245 minutes, and US $156 [15]. Suzuki and colleagues’ study [16] used principal component analysis and cluster analysis to identify countries in Asia and Africa with high potential for telemedicine development, such as Algeria, Egypt, Morocco, and Indonesia. It concluded that telemedicine could address the scarcity of medical resources in these countries.

Despite the great potential of telemedicine to enhance health care accessibility, its adoption remains relatively limited [12,17]. Studies reported that although there are over 300,000 mobile health (mHealth) apps, the user adoption of mHealth apps is low [18,19]. In China, statistics show that telemedicine services account for only 2% of total outpatient services, indicating the underuse of telemedicine services [10]. Except for technology-specific barriers [17,20], a significant factor contributing to this issue is the insufficient communication between patients and service providers, especially on telemedicine platforms where patients or users must initially visit to use these services [21]. Rosler [22] argues that intentional communication skills and tactics can overcome potential barriers to patient engagement within telemedicine and increase patients’ connection with providers. Similarly, Fernández Coves and colleagues’ study [21] revealed that established means of communication were the most prominent facilitators between patients and service providers at the organizational level of telemedicine adoption in primary care settings.

To succeed on telemedicine platforms, HCPs must effectively engage patients by addressing their needs and preferences [23]. Patient engagement refers to the multidimensional experiences that patients engage with their health management, including cognitive (think), emotional (feel), and behavioral (act) subdimensions of enactment [24,25]. Patient engagement is often used interchangeably with patient activation [26], a concept that focuses on the scenario where patients develop an incremental attitude and have cognitive and behavioral participation in their day-to-day health management [25,27,28]. While there are overlaps between these two concepts, patient engagement is seen as a more holistic consideration, which also includes the psychological involvement during patients’ health management situations [25]. In telemedicine settings, patient engagement has been reported to be positively related to high levels of patient satisfaction, improved patient-provider relationships, and increased involvement in health care management [29-33]. For example, in a review study focusing on patient engagement in using hypertension telemedicine tools, Khanijahani et al [34] found that patients’ engagement levels were associated with blood pressure reduction levels, their performance in follow-up consultations, and their interests in recording and monitoring their health data.

Despite the many benefits of patient engagement in telemedicine, current studies pay scant attention to the communication strategies used on telemedicine platforms and how they effectively engage users [23,35]. Costa and Serra [36] conducted one of the few review studies examining how communication influences patient engagement in telemedicine contexts. They found that effective communication serves as a cornerstone for improving patient adherence to treatment, whereas communication barriers, such as language barriers, can hinder patient participation in their own care. However, their review primarily focused on reviewing the general role of communication rather than identifying specific effective communication strategies, and it was limited to the field of chronic wound management. Understanding communication strategies is crucial for maximizing the potential of telemedicine, as effective communication in telemedicine is an essential prerequisite for its success, which not only fosters initial engagement but also maintains trust and cooperation and ensures the continued participation of telemedicine [37]. Specifically, communication in telemedicine with access to HCPs is argued to have high potential to stimulate patient engagement [38,39], which remains a favorable way to improve health care outcomes in telemedicine [40-42].

### Objectives

Given the rapid growth of telemedicine in health care service delivery and the increasing significance of communication strategies for patient engagement in telemedicine systems [23,35,37], this paper aims to identify the communication strategies promoting patient engagement in telemedicine with HCP-patient interactions by conducting a systematic review of the existing telemedicine studies to explore the effective communication strategies discussed. As such, we propose the following research questions (RQs) to guide our study:

RQ1: What communication strategies have been found or hypothesized to contribute to patient engagement on telemedicine platforms with HCP-patient interactions?

RQ2: How has patient engagement in telemedicine been evaluated in the selected literature?

By synthesizing existing research on crucial communication strategies that enhance patient engagement in telemedicine, this review endeavors to provide HCPs, policymakers, telemedicine tool developers, and researchers with insights to inform the development of more effective telehealth strategies and policies.

## Methods

### Overview

This study was conducted following the PRISMA (Preferred Reporting Items for Systematic Reviews and Meta-Analyses) guidelines [43]. We registered this systematic review on PROSPERO (International Prospective Register of Systematic Reviews; CRD420251053245). This study has been revised and updated from the originally registered PROSPERO protocol to incorporate methodological and reporting improvements based on editorial feedback.

### Eligibility Criteria

We included studies if (1) they involved telemedicine using ICTs to deliver health care services, (2) they studied telemedicine tools including HCP-patient interactions, (3) they examined communication strategies influencing patient engagement, (4) they involved real patients or clinical populations who actively engaged with telemedicine, (5) they were peer-reviewed empirical studies, (6) they were published in English, and (7) they were available with full texts.

Articles were excluded if they did not include HCP-patient interactions and only included patients’ health care management functions or health care education information in the telemedicine tool. We excluded studies that used standardized, virtual, or fictional patients without actual patient use with the telemedicine platform, as well as studies that focused on improving patient involvement and engagement in health care research. During the screening process, we excluded articles that were not empirical studies and were not published in a peer-reviewed journal, such as conference papers, editorial notes, and book chapters.

### Search Strategy

We applied the PRISMA-S (Preferred Reporting Items for Systematic reviews and Meta-Analyses literature search extension; Multimedia Appendix 1) to guide our search strategy [44] and searched Web of Science, PubMed, Scopus, MEDLINE (via EbscoHost), CINAHL (via EbscoHost), and Embase for relevant studies because these databases ensure that researchers can find comprehensive studies in a wide range of disciplines, including medicine, public health, and social sciences [45-49]. Two experienced librarians specializing in health, social science, and humanities provided professional consultation to help refine and enhance our search strategy. We summarized and searched key terms of “telemedicine,” “patient engagement,” and “HCP-patient interaction” in the title or abstract, or keywords as shown in Textbox 1. The search strategy combined these three concept blocks using Boolean operators (search strategy: Category 1 AND Category 2 AND Category 3). Apart from using three groups of key terms to identify relevant literature, no language or other restrictions were applied to the search, which was completed on October 31, 2025. The full research strategies applied to the 6 databases are summarized in Multimedia Appendix 2.

**Key terms and search strategy for studies on communication strategies influencing patient engagement in telemedicine involving health care provider (HCP)–patient interactions.**

**Category 1: telemedicine**

eHealth OR e-health OR “electronic health” OR e-consultation OR econsultation* OR e-therapy OR mHealth OR “mobile health” OR telecare OR “tele care” OR telecardiology OR teleconsultation* OR teledentistry OR teledermatology OR telediagnosis OR telehealth OR “tele intensive care” OR “tele ICU” OR telemedicine OR telemonitoring OR telenephrology OR teleneurology OR telenursing OR telepathology OR telepharmacy OR telepsychiatry OR teleradiology OR teleradiotherapy OR telerehabilitation* OR tele-referral* OR “tele referral*” OR telesurgery OR teletherapy OR “virtual care” OR “remote care” OR “virtual medicine” OR “remote rehabilitation*” or “virtual rehabilitation*”

**Category 2: patient engagement**

“patient activation” OR “patient-centeredness” OR “patient engagement” OR “patient involvement” OR “patient participation”

**Category 3: HCP-patient interaction**

consultation* OR “online consultation*” OR “video consultation*” OR “video visit*” OR “virtual visit*” OR “remote visit*” OR “televisit*” OR “virtual appointment*” OR “remote appointment*” OR “clinician-patient interaction*” OR “clinician-patient communication*” OR “doctor-patient interaction*” OR “doctor-patient communication” OR “provider-patient interaction*” OR “provider-patient communication” OR “patient-provider interaction*” OR “patient-provider communication” OR “healthcare professional-patient communication” OR “healthcare professional-patient interaction*” OR “HCP-patient interaction*” OR “HCP-patient communication”

### Selection Process

A total of 3 authors participated in the selection process. After removing the duplicates, the first reviewer (YH) and the second reviewer (RJ) independently screened all titles and abstracts for eligibility. Any discrepancies regarding study eligibility were resolved through discussion with a third reviewer (CSBN), who served as the adjudicator and made the final decision. During the full-text screening phase, the first reviewer (YH) and second reviewer (RJ) independently assessed all studies, and any disagreements were again resolved in consultation with the third reviewer (CSBN).

### Data Collection Process

After the selection process, 2 reviewers (YH and RJ) independently extracted data from each included study using a standardized data extraction table [50] developed for this review. The extraction form was piloted on 7 studies to ensure clarity and consistency. Extracted data included reference, study setting, country, type and number of participants, recruitment and sampling of participants, participant characteristics, enrollment time, telemedicine type, communication strategies influencing patient engagement, and patient engagement measures. Any discrepancies between reviewers were resolved through discussion. The data extraction table is presented in Multimedia Appendix 3 [39,51-83].

### Study Outcomes

The primary outcome domains for this review were (1) communication strategies influencing patient engagement in telemedicine, and (2) methods used to evaluate patient engagement. Communication strategies were defined as any provider-, team-, or system-level communicative actions or decisions intended to enhance communicative effectiveness or compensate for communicative barriers [84-86], thereby shaping patients’ cognitive, emotional, or behavioral engagement [25] during telemedicine encounters. Patient engagement measure was defined as any qualitative or quantitative approaches used to assess patients’ cognitive, emotional, or behavioral engagement in telemedicine. All results that were compatible with these outcome domains were extracted regardless of the time frame of measurement.

The secondary outcomes extracted from each study included reference information, study setting, country, type and number of participants, recruitment and sampling of participants, participant characteristics, enrollment time, and telemedicine type. The extracted information provided contextual information necessary for interpreting outcome variability across studies.

### Quality Assessment

The critical appraisal tool, Mixed Methods Appraisal Tool (MMAT), was used to assess the quality of research included in this study [87]. This tool provides a flexible framework for appraising qualitative, quantitative, and mixed methods studies included in a systematic review [87]. The first reviewer (YH) and the second reviewer (RJ) appraised all the included studies in quality assessment independently, and any disagreements were discussed and resolved with the third reviewer (CSBN) [88]. The product of the quality assessment can be found in the Methodological Quality subsection in the Results section.

### Synthesis Methods

We conducted a deductive and inductive qualitative content analysis [89-91] to identify and analyze words, phrases, and texts extracted in the critical primary outcome domain, that is, the communication strategies influencing patient engagement. The extracted content was then examined through thematic analysis to develop sub-themes and overarching themes representing different types of communication strategies. Approaches used to assess patient engagement were also summarized.

An initial codebook for coding the primary outcome domains was developed based on 10 included studies, and new codes were added inductively as the analysis progressed. Multiple coding approaches were applied to ensure comprehensive analysis, since multicoding helps to reveal patterns and associations within the data, providing deeper insights [92,93]. The coding was conducted by two researchers, both with backgrounds in health communication and content analysis methodologies. The first coder (YH) and the second coder (RJ) performed 20% of the initial coding independently. The intercoder reliability was calculated using Cohen κ. The resulting κ=0.82 indicated almost perfect agreement [94]. The first coder (YH) then coded the rest of the included articles. Finally, the third coder (CSBN) reviewed a portion (4/34, 11.76%) of studies to further assess coding accuracy and ensure consistency. Any discrepancies were discussed and resolved through consensus.

A meta-analysis was not performed due to substantial methodological and contextual heterogeneity across studies. Meta-analysis requires sufficient homogeneity in study design, population, intervention, and outcome measures to ensure meaningful comparability of effect estimates [95]. Given the wide variation in health care contexts, forms of telemedicine, research methods, participant groups, as well as the limited number of comparable quantitative findings in the included research, a narrative synthesis was conducted instead. Consequently, quantitative effect measures (eg, risk ratios, odds ratios, and mean differences), methods to explore statistical heterogeneity (eg, subgroup analysis and meta-regression), sensitivity analyses, assessment of reporting bias due to missing results, and certainty or confidence assessment were not performed, as this review did not aim to statistically pool outcomes across studies. This synthesis approach emphasized thematic patterns in communication strategies and their reported influence on patient engagement.

## Results

### Study Selection and Study Characteristics

**Figure 1:**
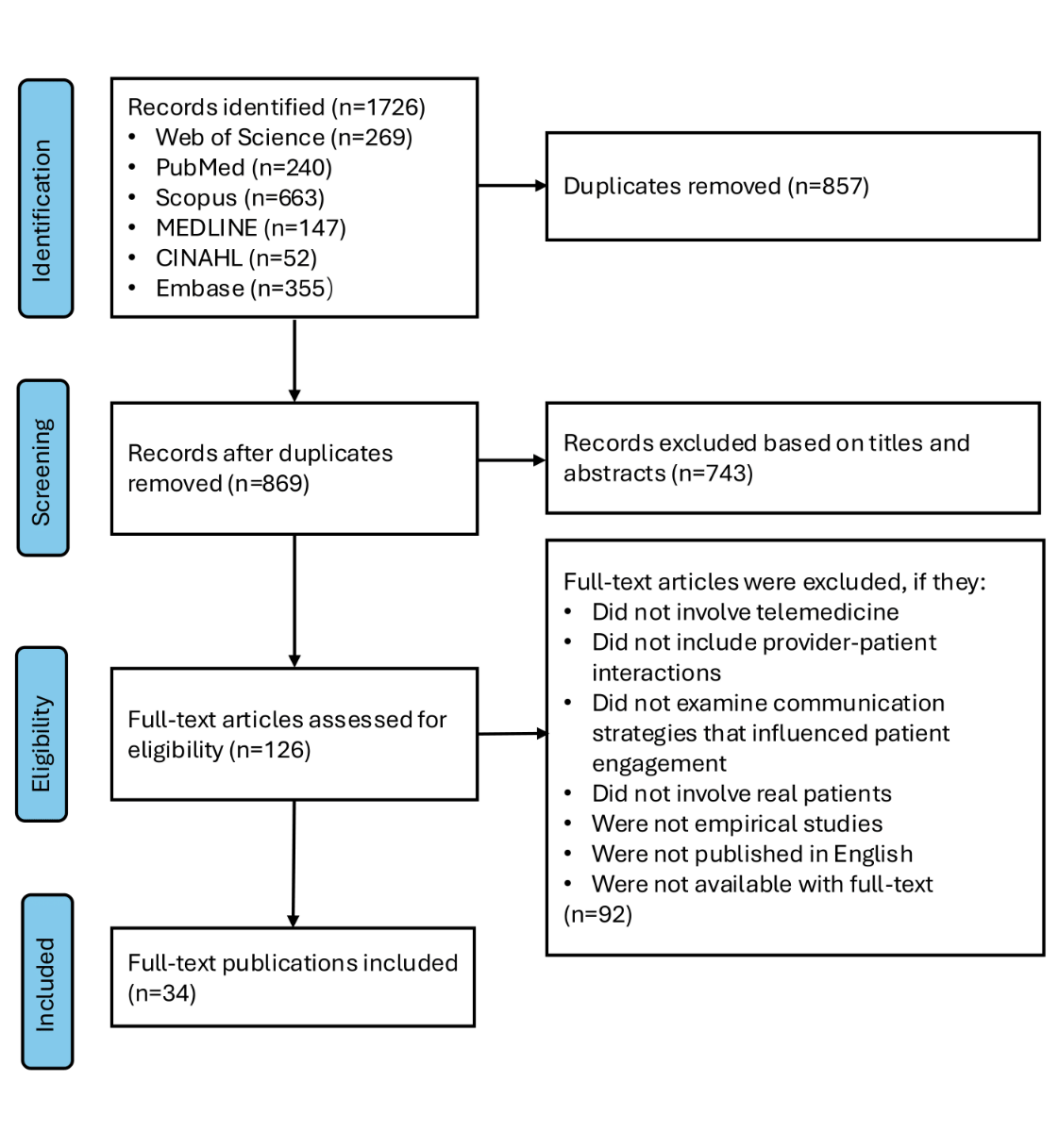
Flowchart of the literature search and screening process for studies on communication strategies influencing patient engagement in telemedicine involving health care provider–patient interactions (1998-2025).

In total, 1726 articles were retrieved from 6 identified databases: Web of Science (n=269), PubMed (n=240), Scopus (n=663), MEDLINE (n=147), CINAHL (n=52), Embase (n=355). These studies were published between 1998 and 2025. After removing 857 duplicates, 869 studies remained to review titles and abstracts, and 126 studies were identified as potentially relevant documents. After the full-text review, 34 studies [39,51-83] were included in this systematic review (Figure 1). Included studies were published between 2015 and 2025, with 28/34 (82.35%) articles published after 2020, reflecting a growing scholarly focus on communication processes within rapidly evolving telemedicine practices. A list of included studies is provided in Multimedia Appendix 3 [39,51-83], and Table 1 presents primary outcomes of data extraction [50].

**Table 1:** Primary outcomes of data extraction on communication strategies influencing patient engagement and patient engagement measures.

Reference, year	Communication strategies influencing patient engagement	Patient engagement measures
Ackerman et al [51], 2020	Trust-based communication between patients and primary care clinicians; Using understandable language to provide clear explanations and updates about eConsult decisions.	No standardized measure; patient engagement was reflected qualitatively via patient accounts of their acceptability of eConsult and feeling involved in care decisions.
Alpert et al [52], 2022	Using a sincere, empathetic tone and plain language to communicate with patients; Offering emotional support; Encouraging patients’ participation by using open-ended questions, validating patient input, and fostering dialogue; Responding promptly to convey accessibility and approachability.	No standardized measure; patient engagement was reflected qualitatively via clinician accounts of patients’ participation, emotional responsiveness, and message interactivity.
Bavngaard et al [53], 2023	Use of visuality in surroundings, such as showing the medicine bottles, facilitated communication; Nonverbal communication through gaze direction and smartphone positioning signaled attentiveness and engagement; Patients’ gaze disengagement was interpreted as cognitive engagement in decision-making; Showing rapport by permitting gaze disengagement from patients.	No standardized measure; patient engagement was operationalized through the observation and thematic analysis of eight video-recorded consultations, focusing on exploring patients’ verbal and non-verbal actions, including attending, contributing, clarifying, and signaling attentiveness.
Björndell [54], 2021	Listening to patients’ thoughts, concerns, and requests; Guiding and trusting patients in self-examination during video consultations.	No standardized measure; patient engagement was reflected qualitatively via physicians’ accounts of patients’ active participation in the consultation process, and patient involvement in decision-making.
Breton et al [55], 2021	Using visual cues, such as seeing patients’ facial expressions, during video visits to enhance communication; Avoiding the issue of reduced confidentiality of consultations, such as conducting consultations with patients during the patient’s grocery time.	No standardized measure; patient engagement was reflected qualitatively via physicians’ perceptions of patients’ access, participation, and responsiveness during telemedicine consultations, including comfort, comprehension, follow-up adherence, and involvement in decision-making.
Brodar et al [56], 2022	Teamwork between departments, including joint virtual visits, interdisciplinary “warm handoffs” between endocrinologists and psychology staff during virtual visits, educating the importance and relevance of consultation and care, and sharing important documents in electronic health records; Encouraging the provider team to use creative and interactive methods to engage patients, such as playing an online game, using the Zoom Whiteboard feature, and sharing the screen to review materials; Ensuring staff training in telemedicine.	No standardized measure; patient engagement was recorded based on the psychosocial screener completion and consultation rates, as well as reflected qualitatively via team members’ feedback about patient acceptability of online consults and patient participation.
Caffery et al [57], 2017	Confusion around issues such as medical liability, privacy, and storage of images was identified as a barrier to patient engagement; Communication issues, such as a language barrier, between the clinicians and patients hindered engagement.	No standardized measure; patient engagement was reflected qualitatively via practitioners’ perceptions of patient satisfaction, participation in teleconsultations, and continuity of care.
Davoust et al [58], 2025	Building rapport and trust through open, honest communication; Visual connections with providers; Providing tailored communication, such as flexibility in visit modalities to accommodate patient preferences.	No standardized measure; patient engagement was assessed qualitatively through participants’ narratives about their experiences and perceived patient involvement in care.
Day et al [59], 2025	A consistent, thorough, and mechanistic consultation structure helped engage patients; Usefulness of information, such as appointment and treatment reminders, increased engagement.	No standardized measure; patient engagement was reflected qualitatively through semistructured interviews.
Dong et al [60], 2023	Established clinician-patient relationships influenced engagement.	No standardized measure; patient engagement was assessed through self-report surveys on patients’ engagement with tele-mental health sessions.
Esayed et al [61], 2025	Building rapport through prior in-person contact and avoiding impersonal communication in telemedicine; Providing interactive communication through facilitating dialogue and openness.	No standardized measure; patient engagement was reflected qualitatively through patients’ perceptions of their preference for telecare.
Gibson et al [62], 2016	Interactive communication, such as speaking directly with consultants and getting feedback from them.	No standardized measure; patient engagement was reflected qualitatively through patient accounts of their involvement in the process of teleconsultations and decision-making.
Grens et al [63], 2022	Concerns about missing nonverbal cues in video consultations; Concerns about impersonal telemedicine visits.	No standardized measure; patient engagement was reflected qualitatively through participant accounts of their involvement in the process of teleconsultations.
Grove et al [64], 2023	Providing feedback on patient-reported outcomes enhanced patient engagement; The opportunity to initiate dialogue with providers; Taking patients seriously and making them feel seen.	No standardized measure; patient engagement was reflected qualitatively based on patients’ perceptions and experiences, such as feelings of support, understanding of their condition, willingness to share information, and overall satisfaction with the remote follow-up.
Higa et al [65], 2021	Tailoring communication style to meet patient preference; Building trust-based relationships with patients, such as encouraging patients through text messaging.	No standardized measure; patient engagement was reflected qualitatively based on multiple data sources, including participants’ feedback interview data, answers to open-ended survey questions, the lead researcher’s participant observations, and field notes from group meetings, telehealth sessions, and informal interactions with participants, text messages, emails, etc. Engagement outcomes included improvements in diabetes knowledge, frequency of blood glucose monitoring, self-care behaviors, and hemoglobin A1c levels.
Islind et al [66], 2019	Interactive dialogic loop based on text and links shared via a text chat; Explaining the reason why health care providers shifted sight and lost eye contact due to screen changes; Understanding, acknowledging, caring, and trusting patients.	No standardized measure; patient engagement was reflected qualitatively based on interview data and the researcher’s observations.
James et al [67], 2021	Concerns about missing nonverbal cues during teleconsultations; Considering patients’ multicultural backgrounds and allowing them to bring interpreters to facilitate communication.	No standardized measure; patient engagement was reflected qualitatively based on nurses’ perceptions of patients’ need to be seen and respected with cultural sensitivity.
Jensen et al [68], 2023	Establishing relationships with patients to engage in meaningful conversations.	No standardized measure; patient engagement was reflected qualitatively through patient accounts of their engagement in care.
Jethwa et al [69], 2022	Establishing relationships between patients and providers to engage in meaningful conversations; Having trust and building rapport; Concerns from patients who do not speak English as a first language; Ensuring clarity in layman’s terms; Being emphatic when communicating with patients.	No standardized measure; patient engagement was reflected qualitatively through answers to open-ended questions in a questionnaire, collecting patient preferences for telemedicine.
Jung et al [70], 2023	Increasing interactions with patients to enhance both patient and staff engagement.	No standardized measure; patient engagement was primarily observed through participation in daily symptom reporting via mobile/web apps and nurse call follow-ups.
Moore et al [71], 2022	Prompt responses from providers to show care; Maintaining established, ongoing patient-provider relationships to foster trust; Provider knowledge and support regarding portal features; Useful functions, such as written records to facilitate communication and engagement; The user-friendly design impacted patients’ decisions about how or to what extent they used the portal; Concerns about the security of the portal.	No standardized measure; patient engagement was assessed qualitatively through patients’ perceptions of their willingness to use telemedicine tools and their preferences for these tools.
Morrison et al [72], 2021	Ease of use regarding Near Me facilitated continued use of this tool.	No standardized measure; patient engagement was assessed through feedback collected via surveys, informal verbal feedback during appointments, and participation in improvement cycles, contributing to iterative service refinement.
Olayiwola et al [73], 2018	Establishing a trust-based provider-patient relationship; Ensuring responsibilities and roles between clinicians were clearly communicated to patients; Providing patients with clear explanations of referral processes and allowing communication for clarifications; Coordination and communication between health care departments; Cultural-linguistic alignment facilitated acceptance of the electronic consultation and referral; Potential security and confidentiality concerns hindered engagement.	No standardized measure; patient engagement was assessed qualitatively through focus groups and survey responses, focusing on perceptions, preferences, and attitudes toward involvement in electronic referral processes.
Osmundsen et al [74], 2015	Increased knowledge and understanding of patients’ disease improved patient engagement.	No standardized measure; patient engagement was assessed qualitatively through participant questionnaires and interviews, focusing on perceptions of care involvement.
Rodkjær et al [75], 2022	Using the information patients provide to increase patient engagement and focus on patients’ needs.	No standardized measure; patient engagement was assessed qualitatively through participant questionnaires and interviews, focusing on their perceptions of patient involvement in remote care.
Scruton et al [76], 2025	Smooth communication between multiple health care providers; Forming trusting and strong physician-patient relationships; Giving patients time to process information and ask questions; Providing emotional support; Including useful functions or information, such as designing straightforward processes to obtain information, support, and care.	No standardized measure; patient engagement was assessed qualitatively through patient perceptions of engagement, specifically feeling cared for and their desire to continue virtual options post pandemic.
Selick et al [77], 2023	Using visual aids and assistive communication tools, choosing appropriate modalities (video over telephone) to support visual and nonverbal cues; Using nonverbal communication, including body language and facial expressions, to support patient comprehension; Establishing connections and building trusting relationships with providers.	No standardized measure; patient engagement was assessed qualitatively based on participant reports of participation, comfort, and involvement during virtual encounters.
Spiess et al [78], 2023	Concerns about “virtual inhibition,” such as missing nonverbal cues and expressing empathy virtually to engage patients from the perspective of providers; Using artwork to start a conversation and connect with patients meaningfully, helping them feel safe for self-disclosure.	No standardized measure; patient engagement was primarily assessed through providers’ perceptions of patient participation, such as self-disclosure, during virtual visits.
Trondsen et al [79], 2018	Facilitating immediacy of assessment through real-time visual and verbal interaction; Building trusting relationships; Providing a sense of access to the “real” expert (psychiatrist), making patients feel seen and heard and invited to decision-making; Showing professionalism in clearly justifying and clarifying assumptions and expectations.	No standardized measure; patient engagement was qualitatively assessed based on participants’ perceptions of patient involvement, the sense of being seen and heard, and the feeling of being involved in decision-making during video consultations.
Van Middelaar et al [39], 2018	Building trusting relationships; Providing useful information or functions, such as personal reminder and the measurement functionality; User-friendliness design, such as the clear layout; Timely and adequate response; Using a positive and personal tone to motivate the use of telemedicine tools.	No standardized measure; patient engagement was assessed qualitatively through interview themes addressing initial and sustained use and perceived usability.
Wei and Mao [80], 2023	Using small talk; Establishing doctor-patient connections.	No standardized measure; patient engagement was assessed qualitatively through the analysis of patients’ interactional behavior, such as initiation, avoidance, refusal, and topic shifting in the conversation excerpts.
White et al [81], 2024	Asking questions and encouraging patient participation; Providing clear explanations and checking for understanding; Using visual aids; Interactive communication, such as screen sharing (for video consultations) and sending links or additional resources, during the consultation; Clarifying information and summarizing key points, engaging patients with health knowledge; Using small talk to build rapport; Building trusting relationships.	No standardized measure; patient engagement was assessed by using multiple research methods, including discourse analysis and conversational analysis to study telehealth consultation recordings, interviewing patients and providers, and conducting patient surveys by asking patients to rate the engagement questions.
Wood et al [82], 2021	Concerns about diminished rapport from clinicians.	No standardized measure; patient engagement was primarily assessed qualitatively through participants’ perceptions of engagement during telehealth visits.
Zainal et al [83], 2024	Clear explanation of medical conditions and treatments, and doctors’ efficiency was appreciated; Maintaining eye contact during consultations (valued but not essential in telehealth); Empathy and respectful communication, and doctors’ abilities to address patient concerns patiently and compassionately.	No standardized measure; patient engagement was assessed qualitatively based on participants’ perceptions of patient involvement and participation during consultations.

### Methodological Quality

The methodological quality assessment using the MMAT indicated generally high quality across the 34 included studies [39,51-83]. Of these, 22 used qualitative designs and 12 used mixed methods approaches. All studies presented clear research questions or objectives, and the collected data were appropriate for addressing them. Overall, the included studies demonstrated a low risk of bias. A summary of the quality assessment is provided in Table 2.

**Table 2:** Quality assessment of included studies on communication strategies influencing patient engagement in telemedicine with health care provider–patient interactions using the Mixed Methods Appraisal Tool.

Reference	Year of Publication	All studies			Qualitative studies						Mixed methods				
		S1^a^	S2^b^	1.1^c^		1.2^d^	1.3^e^	1.4^f^	1.5^g^	5.1^h^		5.2^i^	5.3^j^	5.4^k^	5.5^l^
Ackerman et al [51]	2020	✓	✓	✓		✓	✓	✓	✓						
Alpert et al [52]	2022	✓	✓	✓		✓	✓	✓	✓						
Bavngaard et al [53]	2023	✓	✓	✓		✓	✓	✓	✓						
Björndell et al [54]	2021	✓	✓	✓		✓	✓	✓	✓						
Breton et al [55]	2021	✓	✓	✓		✓	✓	✓	✓						
Brodar et al [56]	2022	✓	✓							✓		✓	✓	✓	✓
Caffery et al [57]	2017	✓	✓	✓		✓	✓	✓	✓						
Davoust et al [58]	2025	✓	✓	✓		✓	✓	✓	✓						
Day et al [59]	2025	✓	✓							✓		✓	✓	C^m^	✓
Dong et al [60]	2023	✓	✓							✓		✓	✓	C	✓
Esayed et al [61]	2025	✓	✓	✓		✓	✓	✓	✓						
Gibson et al [62]	2016	✓	✓	✓		✓	✓	✓	✓						
Grens et al [63]	2022	✓	✓							✓		✓	✓	✓	✓
Grove et al [64]	2023	✓	✓	✓		✓	✓	✓	✓						
Higa et al [65]	2021	✓	✓							✓		✓	✓	✓	✓
Islind et al [66]	2025	✓	✓	✓		✓	✓	✓	✓						
James et al [67]	2021	✓	✓	✓		✓	✓	✓	✓						
Jensen et al [68]	2023	✓	✓	✓		✓	✓	✓	✓						
Jethwa et al [69]	2023	✓	✓	✓		✓	✓	✓	✓						
Jung et al [70]	2022	✓	✓							✓		✓	✓	✓	✓
Moore et al [71]	2022	✓	✓	✓		✓	✓	✓	✓						
Morrison et al [72]	2021	✓	✓							✓		✓	✓	✓	✓
Olayiwola et al [73]	2018	✓	✓							✓		✓	✓	✓	✓
Osmundsen et al [74]	2015	✓	✓	✓		✓	✓	✓	✓						
Rodkjær et al [75]	2022	✓	✓							✓		✓	✓	✓	✓
Scruton et al [76]	2023	✓	✓	✓		✓	✓	✓	✓						
Selick et al [77]	2025	✓	✓							✓		✓	✓	✓	✓
Spiess et al [78]	2023	✓	✓	✓		✓	✓	✓	✓						
Trondsen et al [79]	2018	✓	✓	✓		✓	✓	✓	✓						
Van Middelaar et al [39]	2018	✓	✓	✓		✓	✓	✓	✓						
Wei and Mao [80]	2023	✓	✓	✓		✓	✓	✓	✓						
White et al [81]	2024	✓	✓							✓		✓	✓	✓	✓
Wood et al [82]	2021	✓	✓							✓		✓	✓	✓	✓
Zainal et al [83]	2024	✓	✓	✓		✓	✓	✓	✓						

^a^S1: Are there clear research questions?

^b^S2: Do the collected data allow addressing the research questions?

^c^1.1: Is the qualitative approach appropriate to answer the research question?

^d^1.2: Are the qualitative data collection methods adequate to address the research question?

^e^1.3: Are the findings adequately derived from the data?

^f^1.4: Is the interpretation of results sufficiently substantiated by data?

^g^1.5: Is there coherence between qualitative data sources, collection, analysis, and interpretation?

^h^5.1: Is there an adequate rationale for using a mixed methods design to address the research question?

^i^5.2: Are the different components of the study effectively integrated to answer the research question?

^j^5.3: Are the outputs of the integration of qualitative and quantitative components adequately interpreted?

^k^5.4: Are divergences and inconsistencies between quantitative and qualitative results adequately addressed?

^l^5.5: Do the different components of the study adhere to the quality criteria of each tradition of the methods involved?

^m^C: Can’t tell.

### Results of Syntheses

Based on 34 studies [39,51-83] included in this review, 3 themes of communication strategies were identified as associated with patient engagement: “interpersonal communication strategies,” “team-level communication strategies,” and “system-level communication strategies.” Most studies used qualitative methods, including semistructured interviews and focus groups, to collect information about patient engagement. Studies also used mixed methods to collect patient engagement data, such as combining telemedicine tool use data with patients’ qualitative feedback, to understand patient engagement.

### Communication Strategies to Promote Patient Engagement in Telemedicine

**Figure 2:**
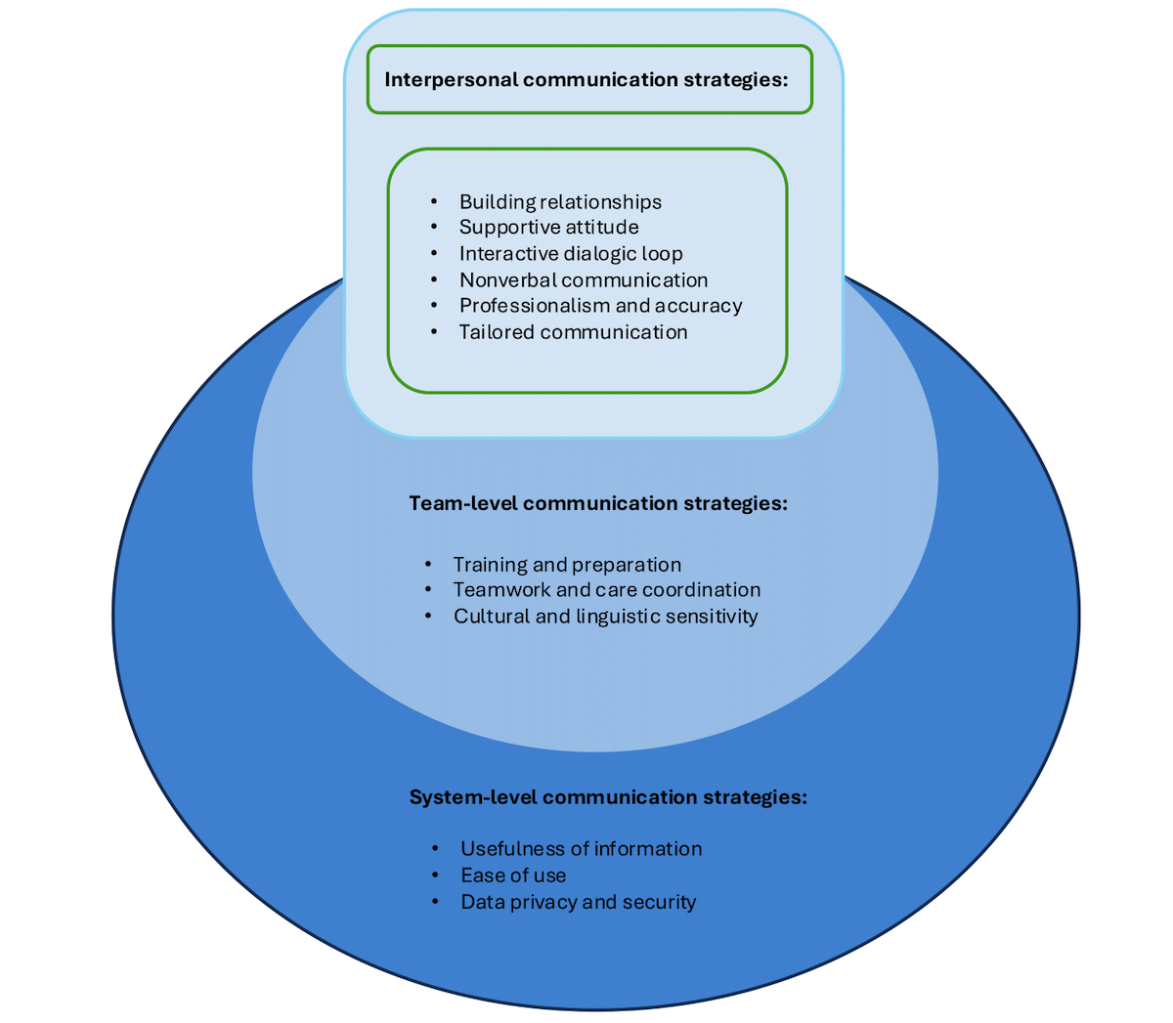
Communication strategies promoting patient engagement in telemedicine, identified in studies involving health care provider–patient interactions across various clinical contexts (2015-2025).

Based on content analysis of included studies, three synthetic constructs were identified and synthesized; that is, interpersonal communication strategies, team-level communication strategies, and system-level communication strategies, which covered micro-, meso-, and macrolevels of communication strategies to enhance patient engagement in the environment of telemedicine. We developed Figure 2 to illustrate the conceptual framework, with the subsequent content explaining how the 3 levels of communication strategies contribute to patient engagement in telemedicine.

### Microlevel: Interpersonal Communication Strategies

At the microlevel, included studies presented prominent interpersonal communication strategies in direct HCP-patient interactions that could improve patient engagement in telemedicine. Specifically, we synthesized 6 subthemes at this level, including building relationships, supportive attitude, interactive dialogic loop, nonverbal communication, professionalism and accuracy, and tailored communication.

The majority of included studies argued that building relationships between HCPs and patients positively impacted patient engagement on diverse telemedicine platforms. Studies found that when patients developed positive and trusting relationships with clinicians, nurses, or other HCPs, they were more likely to accept telemedicine, engage in meaningful conversations with providers, and complete consultation tasks [51,68,71,79,81]. Interpersonal relationship was not only the prerequisite for patients to share their health behaviors, medical concerns, and potential goals [39,78], but also the necessary condition to sustain engagement with telemedicine tools [39]. On the contrary, without established HCP-patient relationships, patients might have concerns about impersonal telemedicine visits [61,63]. Positive interpersonal relationships with HCPs could be built through previous in-person visits [61], visual cues during teleconsultations, such as seeing patients’ facial expressions [54,55].

The second subtheme in interpersonal communication strategy is supportive attitude. During teleconsultations, providers were expected to demonstrate a supportive and sincere approach to enhance patient engagement [39,52]. When patients discussed their health behaviors, providers needed to take them seriously, actively listen, understand their concerns, and acknowledge their challenges [54,64,66]. Effective communication to engage patients also involved incorporating “emotional content,” such as showing care [71,79], expressing empathy [52,83], and praising patients for positive health behaviors [76]. Such supportive attitudes and actions enabled patients to perceive rapport and genuine support from HCPs [52,79,82], which in turn encouraged greater participation in teleconsultations.

An interactive dialogic loop between HCPs and patients was identified as a crucial component of interpersonal communication strategies that enhanced patient engagement in telemedicine. Direct two-way communication with providers not only strengthened patients’ cognitive engagement, such as improving their understanding of disease and increasing access to health knowledge [62,74,81], but also promoted behavioral engagement by encouraging active participation in treatment [52]. During teleconsultations, providers were expected to use a range of communication skills to sustain dialogue and foster engagement [52,61]. These included having small talk [80,81], finding common topics such as artwork to start a conversation and connect with patients [78], explaining the underlying causes of symptoms in detail [52,62], asking open-ended questions [52,53], checking patients’ understanding [81], giving patients time to ask questions [76,81], and using chat functions to share screens and links for interactive exchanges [66,81]. In asynchronous communication, prompt and adequate responses to patient messages were essential for stimulating patient engagement, as patients felt reassured by sufficient access to HCPs [39,52]. Conversely, delays or lack of responses often led patients to discontinue platform use [39,64]. Across both synchronous and asynchronous consultations, clear and accessible communication in lay terms was consistently reported to encourage dialogues and strengthen provider-patient interactions [51,52,69].

Nonverbal communication was also found to play a critical role in patient engagement in telemedicine [77]. Studies noted that patients were concerned about the lack of nonverbal cues, such as being able to see what doctors were doing during telephone consultations [67] or missing body language during video consultations [63]. Islind et al [66] and Bavngaard et al [53] further highlighted the role of eye gaze in shaping patient engagement during teleconsultations. Islind et al [66] emphasized that explaining the reason why HCPs shifted their gaze or lost eye contact, often due to screen changes, was important for sustaining engagement. On the other hand, Bavngaard et al [53] underscored the value of allowing flexibility in patients’ gaze directionality and even acknowledging momentary gaze disengagement, as brief breaks in eye contact could signal thoughtful and active involvement during consultations. They also highlighted that leveraging visual elements in the surroundings, such as showing the medicine bottles to convey accurate information, could facilitate patients’ active participation [53]. Taken together, body language, eye gaze, and the use of visual objects were identified as key nonverbal communication strategies associated with patient engagement.

Within interpersonal communication strategies in telemedicine, patients emphasized the importance of both professionalism and accuracy, as well as tailored communication from HCPs. Zainal et al [83] found that although patients appreciated eye contact during teleconsultations, they placed great value on providers’ efficiency and accuracy in communication to avoid errors. Conversely, when providers failed to justify or clearly clarify assumptions and expectations during teleconsultations, patient disengagement was evident [79]. Higa et al [65] highlighted that adapting communication according to patients’ individual preferences was crucial for sustaining their engagement. For instance, while some patients responded positively to providers who gave nurturing and encouraging suggestions, others preferred a strict and relentless communication style. Similarly, Davoust et al [58] found that although patients valued a trusting relationship and positive rapport, their levels of comfort varied. Therefore, offering patients flexible options and implementing tailored approaches in telemedicine are essential to accommodate individual preferences and needs.

### Mesolevel: Team-Level Communication Strategies

Included studies in this review also presented how communication strategies used by health care teams and organizations could influence patient engagement. A total of 3 subthemes, that is, training and preparation, teamwork and care coordination, and cultural and linguistic sensitivity in health care teams, were synthesized from the mesolevel of communication strategies in telemedicine.

Training and preparation in HCP teams was identified as crucial for patient engagement in telemedicine [56,73]. Patients who experienced difficulty in sustaining attention or “Zoom fatigue” during a remote visit might reduce engagement. To solve this issue, health care organizations should ensure that providers receive communication training in telemedicine, such as using the screen-sharing function to engage patients and playing an online game [56]. Members in provider teams should prepare and provide consistent and clear explanations of the teleconsultation process with patients to have their questions answered, which was reported to impact patients’ acceptance of telemedicine tools [73]. Importantly, preparation in HCP teams extended beyond communication training to necessary patient education, particularly around confidentiality. Patients needed guidance on when and how to participate in teleconsultations appropriately, such as avoiding virtual meetings while at the grocery store or driving, so as to maintain privacy and reduce distraction and disengagement [55].

Teamwork and care coordination were identified as essential to influence patients’ acceptance and use of telemedicine when they received care from multiple providers. Olayiwola et al [73] reported that clearly defined responsibilities and effective coordination among clinicians were prerequisites for patient acceptance of telemedicine. Similarly, Brodar et al [56] found that teamwork across departments and HCPs, such as joint virtual visits, warm handoff through visit summaries, and sharing key information in electronic health records, helped ensure continuity of care and strengthened patient engagement. Conversely, poor communication among multiple HCPs undermined continuity and reduced care quality, leaving patients feeling neglected and less willing to engage in teleconsultations [76].

For patients from multicultural backgrounds, cultural and linguistic sensitivity within health care teams was crucial to alleviating concerns about using telemedicine [57,67,69,73]. Teams needed to recognize potential cultural and language barriers, particularly when providers interacted with patients who were nonnative English speakers [57,69]. In such cases, involving interpreters during teleconsultations was recommended to help overcome these barriers and support patient engagement [67].

### Macrolevel: System-Level Communication Strategies

In addition to identifying communication strategies involving individual HCPs and their teams, this review also examined system-level strategies within telemedicine that influenced patient engagement. A total of 3 key subthemes were identified within this category: usefulness of information, ease of use, and data privacy and security.

Patients reported that the perceived usefulness of information provided by telemedicine platforms, such as self-management tools, personal reminders, access to relevant health information, and a written record function that helped them recall providers’ guidance and details from HCP-patient communication, facilitated their engagement [39,71,76]. Ease of use was another critical system-level factor influencing patients’ adoption and continued use of telemedicine [71,72]. Platforms with a clear and simple layout and user-friendly features increased acceptability [39,71,72], whereas barriers, such as login difficulties, navigation challenges, or app freezing, discouraged patients from ongoing use and reduced the likelihood of recommending telemedicine tools [71].

Additionally, scholars reported that patients were sometimes hesitant to use telemedicine tools due to concerns about data privacy and security [57,71,73]. Given the sensitive nature of personal health information, some patients expressed worry about how their data were stored and protected [57,71]. Therefore, ensuring secure handling and safeguarding patient information on telemedicine platforms is essential to building trust and encouraging patient engagement.

### Evaluation of Patient Engagement

The overwhelming majority of included studies (31/34, 91.18%) used qualitative methods, such as observations, one-on-one interviews, focused groups, asking open-ended questions, and collecting qualitative feedback, to investigate patient engagement from patients and HCPs. Researchers collected qualitative data about patient acceptability of telemedicine, user engagement, patient participation, attention during consultation, and involvement in decision making to evaluate patient engagement. For example, Bavngaard et al [53] conducted a qualitative observational study analyzing 8 video-recorded HCP-patient consultations to explore patient participation during teleconsultations. Van Middelaar et al [39] used semistructured interviews to investigate 20 patients’ engagement experience on an online cardiovascular risk management tool. Olayiwola et al [73] collected patient engagement data from both patient focus groups and HCPs’ perceptions about patient engagement from their open-ended feedback in an online survey.

Three studies [56,60,81] used mixed methods to evaluate patient engagement. Brodar et al [56] combined quantitative components, that is, health screener completion rate and consultation rate as indicators of engagement, with a qualitative component, that is, participants’ feedback through open-ended responses and comments about their telehealth experiences. In Dong and colleagues’ [60] telemental health study, patient engagement was measured through quantitative survey items, such as provider-reported ratings of patient engagement, as well as qualitative feedback from providers’ open-ended responses describing types of patients that engaged or disengaged in tele-mental health services. White et al [81] used multiple research methods to evaluate patient engagement, including using discourse analysis and conversational analysis to study telehealth consultation recordings, interviewing patients and HCPs, and conducting patient surveys by asking patients to rate the engagement questions, which related to the patient’s ability and comfort in communicating and participating in their care from the Telehealth Usability Questionnaire.

## Discussion

### Principal Findings

The objective of this systematic review was to identify communication strategies that influence patient engagement in telemedicine with the function of HCP-patient interactions. A total of 34 peer-reviewed studies were analyzed, revealing 3 overarching themes of effective communication strategies that enhance patient engagement: interpersonal communication strategies, with 6 subthemes (building relationships, supportive attitude, interactive dialogic loop, nonverbal communication, professionalism and accuracy, and tailored communication); team-level communication strategies, with 3 subthemes (training and preparation, teamwork and care coordination, and cultural and linguistic sensitivity); and system-level communication strategies, with 3 subthemes (usefulness of information, ease of use, and data privacy and security). Furthermore, this review found that qualitative research methods were the most commonly employed approach for assessing patient engagement in the included studies.

### Implications Across Micro-, Meso-, and Macrolevel Communication Strategies

At the microlevel, interpersonal communication strategies between HCPs and patients emerged as a cornerstone of enhancing patient engagement in telemedicine. This finding is consistent with previous health care research. For example, Ngai et al [89] highlighted that communication strategies such as maintaining an interactive dialogic loop and demonstrating empathy during two-way HCP-patient communication were crucial for engaging users in health care settings. Similarly, Kwame and Petrucka [96] advanced a patient-centered model, arguing that person-centered communication fosters effective communication and contributes to positive health outcomes. Their model emphasized building meaningful relationships with patients, recognizing their concerns and needs, encouraging self-expression, explaining health conditions and care plans clearly, and engaging in empathetic communication—all of which align with the subthemes of interpersonal communication strategies identified in this review. These insights reinforce the approach of patient-centered communication. Rather than focusing solely on completing consultation tasks, HCPs should view patients as unique individuals with distinct care needs and as collaborators in the care process [65,66,83,96]. Such an approach facilitates effective communication and, ultimately, strengthens patient engagement in telemedicine.

This review identified communication strategies applied not only during synchronous or asynchronous consultations, but also in the form of adequate preparation, particularly at the team level. At the mesolevel, 3 key team-level communication strategies were identified, that is, training and preparation, teamwork and care coordination, and cultural and linguistic sensitivity, which resonate with relational coordination theory [97] and cultural competence model [98]. The relational coordination theory is widely discussed in organizational communication, which emphasizes shared goals, shared knowledge, and mutual respect among team members [97]. This aligns with evidence showing that coordinated teamwork, including team-level communication training in the environment of telemedicine, consistent and clear explanations of the teleconsultation processes, warm handoffs, and joint virtual visits, improved telemedicine acceptance and sustained patient engagement [56,73].

In addition, cultural and linguistic sensitivity emerged as a crucial dimension of team-level communication, consistent with the cultural competence model, which proposes a model of care that includes cultural awareness, knowledge, skills, encounters, and desire [98]. This framework underscores the importance of understanding patients’ unique cultural backgrounds and needs, adapting communication styles, addressing language barriers, and involving interpreters where necessary to ensure equitable access and rapport with diverse patient populations [57,67,69,98]. Collectively, these strategies at the team level illustrate that patient engagement in telemedicine is not only an outcome of interpersonal interactions but also the product of well-prepared, well-coordinated, and culturally responsive health care teams.

The identified system-level communication strategies align with previous research on health-related communication on patient engagement. For example, many health communication studies have validated that providing useful content could improve the engagement of the targeted audience [89,99-101]. In addition, Xie and colleagues’ [102] and Vasiloglou and colleagues’ [103] studies reported that ease of use was a critical reason for users to choose a health app. The identified subthemes of usefulness of information and ease of use at the macrolevel resonate with the technology acceptance model, a leading model in technology acceptance, which argues that users’ perceived usefulness and ease of use are primary factors influencing their adoption of new technologies [104].

Moreover, data privacy and security emerged as a critical system-level communication strategy in this review. Given the highly private and sensitive nature of health care data, it is understandable that some patients were reluctant to adopt telemedicine tools due to concerns about confidentiality [105,106]. To address these concerns, telemedicine developers must prioritize robust data protection measures. Suggested strategies include implementing an authentication mechanism [107] and providing patient telehealth “drop-in” kiosks with devices and soundproof space [82].

### Advancing the Evaluation of Patient Engagement in Telemedicine

It is surprising to find that the included studies in this review predominantly used qualitative methods, such as semistructured interviews and qualitative feedback, to collect data about patient engagement. Research primarily using quantitative measurements of patient engagement was missing from the included studies. Although 3 studies [56,60,81] used surveys to collect participants’ ratings of patient engagement-related items, none of the included studies measured patient engagement in the sense of quantifying engagement through standardized scales. In other words, the quantitative assessment tools for evaluating patient engagement were not unified and standardized. This might be due to a significant lack of clarity regarding the definition and conceptualization of patient engagement, as evidenced by the plethora of terms frequently used interchangeably in this field, as well as the lack of assessment instruments [25].

Not identified in this review, but in a worldwide context, the Patient Activation Measurement (PAM) scale [27] is one of the few assessment scales that have been used to evaluate patient engagement in telemedicine [40,108-110]. The PAM scale was developed to quantify patients’ knowledge, skills, and confidence in managing their health [27,111]. However, although the concepts of patient engagement and patient activation overlap, they differ in their conceptual breadth [25]. As discussed earlier, patient engagement represents a multidimensional psychosocial process in which individuals’ cognitive, emotional, and behavioral actions collectively shape how they manage their health. In contrast, patient activation primarily emphasizes the cognitive and behavioral components of this process [25,31]. As such, the PAM scale could not capture the holistic nature of patient engagement. Another widely accepted patient engagement scale is the 5-item Patient Health Engagement (PHE) scale developed by Graffigna and colleagues [25]. The PHE scale assesses patients’ perceived readiness for cognitive, emotional, and behavioral engagement. However, none of the studies included in this review used this instrument. In addition, patient engagement has been measured in previous research using other standardized tools, such as the observing patient involvement in decision making (OPTION) scale for measuring patient involvement [112], the Perceived Involvement in Care Scale [113], and the Patient Participation Scale [114], none of which were applied in the included studies. Nevertheless, these existing instruments hold potential for integration or adaptation to enable more consistent evaluation of engagement outcomes in future telemedicine research. We summarized available standardized tools for assessing patient engagement and their potential adaptations to telemedicine in Multimedia Appendix 4.

### Limitations and Future Directions

This review has some limitations to note: First, it only included telemedicine studies with HCP-patient interactions. Although telemedicine tools with interactive support from providers have great potential to engage patients [38,39], other studies on telemedicine platforms that focus on patient education, health data management, or the dissemination of health-related information may also incorporate additional effective communication strategies that enhance patient engagement, which can be explored in future reviews. Second, the review did not include gray literature, which may have led to the omission of recent developments or emerging trends in the field first reported at conferences. Incorporating conference proceedings in future review could provide a more comprehensive and up-to-date understanding of the field. Third, this review only included peer-reviewed articles published in English, which may have excluded important research published in other languages that explored telemedicine in various contexts. Despite these limitations, this review serves as a foundational step in the field. It is hoped that future research will address these deficits by exploring the topic more comprehensively.

Future research can explore the following directions in studying effective communication strategies for promoting patient engagement with telemedicine tools. First, researchers should further clarify what patient engagement is by providing a rigorous conceptualization and exploring the dimensions of patient engagement, particularly in the telemedicine environment. Currently, studies have tested and collected data on usability, patient acceptability, patient participation, health condition management, and so on, to understand patient engagement. However, what the components of patient engagement are and how to measure them scientifically remain unclear. In addition to using explorative qualitative methods to ask questions about patients’ attitudes and preferences toward telemedicine tools, validated assessment instruments for patient engagement in this field are expected to be developed. Second, future studies should examine and validate the relationships between 12 subthemes across the 3 overarching communication strategy themes identified in this review and patient engagement. Such efforts could contribute to the development of an integrated communication framework that fosters patient engagement with telemedicine tools. In particular, future studies may explore and empirically test the connections between specific communication subthemes and different dimensions of patient engagement. Third, future work can build on this study by exploring additional telemedicine contexts beyond HCP-patient interactions, integrating grey literature and conference proceedings, and including non-English publications to capture more comprehensive evidence, emerging trends, and broader cultural perspectives on communication strategies influencing patient engagement.

### Conclusion

This systematic review underscores the critical role of various communication strategies in enhancing patient engagement in telemedicine with HCP-patient interactions. A total of 3 themes of communication strategies, namely interpersonal (micro), team (meso), and system (macro) level communication strategies, with 12 subthemes, were identified as important factors influencing patient engagement. This review offers an innovative and pioneering effort to systematically synthesize communication strategies that promote patient engagement in telemedicine. Unlike previous reviews that focused on isolated aspects or levels of communication, our review uniquely integrates strategies across all three levels to provide a holistic and comprehensive framework. Theoretically, it advances understanding of how micro-, meso-, and macrolevel communication strategies collectively influence patient engagement, filling a critical gap in existing literature. Practically, it provides actionable guidance for telemedicine developers, health care professionals, and policymakers. The identified strategies offer a comprehensive framework for improving the quality and sustainability of telemedicine practices. In real-world terms, these insights can inform training programs for health care professionals, guide platform design, and support policy initiatives that promote equitable, patient-centered digital care. We also found that the majority of included studies used qualitative research methods to assess patient engagement. Future studies can further explore, validate, and test quantitative methods to evaluate patient engagement and the relationships between different communication strategies and patient engagement in telemedicine.

## Appendix

Multimedia Appendix 1 PRISMA 2020_S checklist.

Multimedia Appendix 2 Databases and search strategies for studies on communication strategies influencing patient engagement in telemedicine involving healthcare provider-patient interactions.

Multimedia Appendix 3 Data extraction table for included studies on communication strategies influencing patient engagement in telemedicine involving healthcare provider-patient interactions.

Multimedia Appendix 4 Summary of standardized instruments for measuring patient engagement and their relevance and potential to be adapted in telemedicine research.

## Abbreviations

HCP: health care provider
mHealth: mobile health
MMAT: Mixed Methods Appraisal Tool
OPTION: observing patient involvement in decision making
PAM: Patient Activation Measurement
PHE: 5-item Patient Health Engagement
PRISMA: Preferred Reporting Items for Systematic Reviews and Meta-Analyses
PRISMA-S: Preferred Reporting Items for Systematic reviews and Meta-Analyses literature search extension
PROSPERO: International Prospective Register of Systematic Reviews
RQ: research question

## Data Availability

All data generated or analyzed during this study are included in this published article and its supplementary files.
