# Supplementary material for: Communication Strategies to Promote Patient Engagement in Telemedicine: Systematic Review
Source: J Med Internet Res. 2026 Jan 21;28:e85456. doi: 10.2196/85456 (PMC12873561; doi:10.2196/85456)
Supplement: Multimedia Appendix 2 [file jmir_v28i1e85456_app2.docx]

**Multimedia Appendix 2**. Databases and search strategies for studies on communication strategies influencing patient engagement in telemedicine involving healthcare provider-patient interactions.

| Database | Search Fields | Search Strategy |
| --- | --- | --- |
| Web of Science | Topic | eHealth OR e-health OR “electronic health” OR e-consultation OR econsultation* OR e-therapy OR mHealth OR “mobile health” OR telecare OR “tele care” OR telecardiology OR teleconsultation* OR teledentistry OR teledermatology OR telediagnosis OR telehealth OR “tele intensive care” OR “tele ICU” OR telemedicine OR telemonitoring OR telenephrology OR teleneurology OR telenursing OR telepathology OR telepharmacy OR telepsychiatry OR teleradiology OR teleradiotherapy OR telerehabilitation* OR tele-referral* OR “tele referral*” OR telesurgery OR teletherapy OR “virtual care” OR “remote care” OR “virtual medicine” OR “remote rehabilitation*” or “virtual rehabilitation*”  AND  “patient activation” OR “patient-centeredness” OR “patient engagement” OR “patient involvement” OR “patient participation”  AND  consultation* OR “online consultation*” OR “video consultation*” OR “video visit*” OR “virtual visit*” OR “remote visit*” OR “televisit*” OR “virtual appointment*” OR “remote appointment*” OR “clinician-patient interaction*” OR “clinician-patient communication*” OR “doctor-patient interaction*” OR “doctor-patient communication” OR “provider-patient interaction*” OR “provider-patient communication” OR “patient-provider interaction*” OR “patient-provider communication” OR “healthcare professional-patient communication” OR “healthcare professional–patient interaction*” OR “HCP-patient interaction*” OR “HCP-patient communication” |
| PubMed | Title/Abstract | ((eHealth[Title/Abstract] OR e-health[Title/Abstract] OR "electronic health"[Title/Abstract] OR e-consultation[Title/Abstract] OR econsultation*[Title/Abstract] OR e-therapy[Title/Abstract] OR mHealth[Title/Abstract] OR "mobile health"[Title/Abstract] OR telecare[Title/Abstract] OR "tele care"[Title/Abstract] OR telecardiology[Title/Abstract] OR teleconsultation*[Title/Abstract] OR teledentistry[Title/Abstract] OR teledermatology[Title/Abstract] OR telediagnosis[Title/Abstract] OR telehealth[Title/Abstract] OR "tele intensive care"[Title/Abstract] OR "tele ICU"[Title/Abstract] OR telemedicine[Title/Abstract] OR telemonitoring[Title/Abstract] OR telenephrology[Title/Abstract] OR teleneurology[Title/Abstract] OR telenursing[Title/Abstract] OR telepathology[Title/Abstract] OR telepharmacy[Title/Abstract] OR telepsychiatry[Title/Abstract] OR teleradiology[Title/Abstract] OR teleradiotherapy[Title/Abstract] OR telerehabilitation*[Title/Abstract] OR tele-referral*[Title/Abstract] OR "tele referral*"[Title/Abstract] OR telesurgery[Title/Abstract] OR teletherapy[Title/Abstract] OR "virtual care"[Title/Abstract] OR "remote care"[Title/Abstract] OR "virtual medicine"[Title/Abstract] OR "remote rehabilitation*"[Title/Abstract] OR "virtual rehabilitation*"[Title/Abstract]) AND ("patient activation"[Title/Abstract] OR "patient-centeredness"[Title/Abstract] OR "patient engagement"[Title/Abstract] OR "patient involvement"[Title/Abstract] OR "patient participation"[Title/Abstract])) AND (consultation*[Title/Abstract] OR "online consultation*"[Title/Abstract] OR "video consultation*"[Title/Abstract] OR "video visit*"[Title/Abstract] OR "virtual visit*"[Title/Abstract] OR "remote visit*"[Title/Abstract] OR "televisit*"[Title/Abstract] OR "virtual appointment*"[Title/Abstract] OR "remote appointment*"[Title/Abstract] OR "clinician-patient interaction*"[Title/Abstract] OR "clinician-patient communication*"[Title/Abstract] OR "doctor-patient interaction*"[Title/Abstract] OR "doctor-patient communication"[Title/Abstract] OR "provider-patient interaction*"[Title/Abstract] OR "provider-patient communication"[Title/Abstract] OR "patient-provider interaction*"[Title/Abstract] OR "patient-provider communication"[Title/Abstract] OR "healthcare professional-patient communication"[Title/Abstract] OR "healthcare professional–patient interaction*"[Title/Abstract] OR "HCP-patient interaction*"[Title/Abstract] OR "HCP-patient communication"[Title/Abstract]) |
| Scopus | Article title, Abstract, Keywords | eHealth OR e-health OR “electronic health” OR e-consultation OR econsultation* OR e-therapy OR mHealth OR “mobile health” OR telecare OR “tele care” OR telecardiology OR teleconsultation* OR teledentistry OR teledermatology OR telediagnosis OR telehealth OR “tele intensive care” OR “tele ICU” OR telemedicine OR telemonitoring OR telenephrology OR teleneurology OR telenursing OR telepathology OR telepharmacy OR telepsychiatry OR teleradiology OR teleradiotherapy OR telerehabilitation* OR tele-referral* OR “tele referral*” OR telesurgery OR teletherapy OR “virtual care” OR “remote care” OR “virtual medicine” OR “remote rehabilitation*” or “virtual rehabilitation*”  AND  “patient activation” OR “patient-centeredness” OR “patient engagement” OR “patient involvement” OR “patient participation”  AND  consultation* OR “online consultation*” OR “video consultation*” OR “video visit*” OR “virtual visit*” OR “remote visit*” OR “televisit*” OR “virtual appointment*” OR “remote appointment*” OR “clinician-patient interaction*” OR “clinician-patient communication*” OR “doctor-patient interaction*” OR “doctor-patient communication” OR “provider-patient interaction*” OR “provider-patient communication” OR “patient-provider interaction*” OR “patient-provider communication” OR “healthcare professional-patient communication” OR “healthcare professional–patient interaction*” OR “HCP-patient interaction*” OR “HCP-patient communication” |
| MEDLINE | Title and Abstract | eHealth OR e-health OR “electronic health” OR e-consultation OR econsultation* OR e-therapy OR mHealth OR “mobile health” OR telecare OR “tele care” OR telecardiology OR teleconsultation* OR teledentistry OR teledermatology OR telediagnosis OR telehealth OR “tele intensive care” OR “tele ICU” OR telemedicine OR telemonitoring OR telenephrology OR teleneurology OR telenursing OR telepathology OR telepharmacy OR telepsychiatry OR teleradiology OR teleradiotherapy OR telerehabilitation* OR tele-referral* OR “tele referral*” OR telesurgery OR teletherapy OR “virtual care” OR “remote care” OR “virtual medicine” OR “remote rehabilitation*” or “virtual rehabilitation*”  AND  “patient activation” OR “patient-centeredness” OR “patient engagement” OR “patient involvement” OR “patient participation”  AND  consultation* OR “online consultation*” OR “video consultation*” OR “video visit*” OR “virtual visit*” OR “remote visit*” OR “televisit*” OR “virtual appointment*” OR “remote appointment*” OR “clinician-patient interaction*” OR “clinician-patient communication*” OR “doctor-patient interaction*” OR “doctor-patient communication” OR “provider-patient interaction*” OR “provider-patient communication” OR “patient-provider interaction*” OR “patient-provider communication” OR “healthcare professional-patient communication” OR “healthcare professional–patient interaction*” OR “HCP-patient interaction*” OR “HCP-patient communication” |
| CINAHL | Title and Abstract | eHealth OR e-health OR “electronic health” OR e-consultation OR econsultation* OR e-therapy OR mHealth OR “mobile health” OR telecare OR “tele care” OR telecardiology OR teleconsultation* OR teledentistry OR teledermatology OR telediagnosis OR telehealth OR “tele intensive care” OR “tele ICU” OR telemedicine OR telemonitoring OR telenephrology OR teleneurology OR telenursing OR telepathology OR telepharmacy OR telepsychiatry OR teleradiology OR teleradiotherapy OR telerehabilitation* OR tele-referral* OR “tele referral*” OR telesurgery OR teletherapy OR “virtual care” OR “remote care” OR “virtual medicine” OR “remote rehabilitation*” or “virtual rehabilitation*”  AND  “patient activation” OR “patient-centeredness” OR “patient engagement” OR “patient involvement” OR “patient participation”  AND  consultation* OR “online consultation*” OR “video consultation*” OR “video visit*” OR “virtual visit*” OR “remote visit*” OR “televisit*” OR “virtual appointment*” OR “remote appointment*” OR “clinician-patient interaction*” OR “clinician-patient communication*” OR “doctor-patient interaction*” OR “doctor-patient communication” OR “provider-patient interaction*” OR “provider-patient communication” OR “patient-provider interaction*” OR “patient-provider communication” OR “healthcare professional-patient communication” OR “healthcare professional–patient interaction*” OR “HCP-patient interaction*” OR “HCP-patient communication” |
| EMBASE | Title, Abstract or Author Keywords | (ehealth:ti,ab,kw OR 'e health':ti,ab,kw OR 'electronic health':ti,ab,kw OR 'e consultation':ti,ab,kw OR econsultation*:ti,ab,kw OR 'e therapy':ti,ab,kw OR mhealth:ti,ab,kw OR 'mobile health':ti,ab,kw OR telecare:ti,ab,kw OR 'tele care':ti,ab,kw OR telecardiology:ti,ab,kw OR teleconsultation*:ti,ab,kw OR teledentistry:ti,ab,kw OR teledermatology:ti,ab,kw OR telediagnosis:ti,ab,kw OR telehealth:ti,ab,kw OR 'tele intensive care':ti,ab,kw OR 'tele icu':ti,ab,kw OR telemedicine:ti,ab,kw OR telemonitoring:ti,ab,kw OR telenephrology:ti,ab,kw OR teleneurology:ti,ab,kw OR telenursing:ti,ab,kw OR telepathology:ti,ab,kw OR telepharmacy:ti,ab,kw OR telepsychiatry:ti,ab,kw OR teleradiology:ti,ab,kw OR teleradiotherapy:ti,ab,kw OR telerehabilitation*:ti,ab,kw OR 'tele referral*':ti,ab,kw OR telesurgery:ti,ab,kw OR teletherapy:ti,ab,kw OR 'virtual care':ti,ab,kw OR 'remote care':ti,ab,kw OR 'virtual medicine':ti,ab,kw OR 'remote rehabilitation*':ti,ab,kw OR 'virtual rehabilitation*':ti,ab,kw) AND ('patient activation':ti,ab,kw OR 'patient-centeredness':ti,ab,kw OR 'patient engagement':ti,ab,kw OR 'patient involvement':ti,ab,kw OR 'patient participation':ti,ab,kw) AND (consultation*:ti,ab,kw OR 'online consultation*':ti,ab,kw OR 'video consultation*':ti,ab,kw OR 'video visit*':ti,ab,kw OR 'virtual visit*':ti,ab,kw OR 'remote visit*':ti,ab,kw OR 'televisit*':ti,ab,kw OR 'virtual appointment*':ti,ab,kw OR 'remote appointment*':ti,ab,kw OR 'clinician-patient interaction*':ti,ab,kw OR 'clinician-patient communication*':ti,ab,kw OR 'doctor-patient interaction*':ti,ab,kw OR 'doctor-patient communication':ti,ab,kw OR 'provider-patient interaction*':ti,ab,kw OR 'provider-patient communication':ti,ab,kw OR 'patient-provider interaction*':ti,ab,kw OR 'patient-provider communication':ti,ab,kw OR 'healthcare professional-patient communication':ti,ab,kw OR 'healthcare professional–patient interaction*':ti,ab,kw OR 'hcp-patient interaction*':ti,ab,kw OR 'hcp-patient communication':ti,ab,kw) |
