# Supplementary material for: Communication Strategies to Promote Patient Engagement in Telemedicine: Systematic Review
Source: J Med Internet Res. 2026 Jan 21;28:e85456. doi: 10.2196/85456 (PMC12873561; doi:10.2196/85456)
Supplement: Multimedia Appendix 4 [file jmir_v28i1e85456_app4.docx]

**Multimedia Appendix 4**. Summary of standardized instruments for measuring patient engagement and their relevance and potential to be adapted in telemedicine research.

| Existing Instrument for Measuring Patient Engagement | Original Context | Measurement Properties and Main Constructs Measured | Relevance and Potential to be Adapted in Telemedicine Research |
| --- | --- | --- | --- |
| Patient Activation Measure (PAM) [1] | The PAM scale is originally developed to assess patient self-management and engagement in chronic disease care in the U.S. | The 22-item scale has four subdimensions: believes active role important, confidence and knowledge to take action, taking action, staying the course under stress. | The PAM scale can be adapted to evaluate whether, and to what extent, telemedicine interventions influence patients’ activation levels focusing on cognitive and behavioral engagement in their care. |
| Short Form of Patient Activation Measure (PAM) [2] | The short form of PAM scale is developed to assess patients’ capabilities and readiness in engaging their chronic disease self-care management in the U.S. | The 13-item unidimensional tool measures self-assess knowledge about chronic conditions, beliefs about illness and medical care, and self-efficacy for self-care. | The short form of PAM scale can be adapted to assess how telemedicine interventions influence patients’ self-efficacy and active role in remote care. |
| Patient Health Engagement (PHE) [3] | The PHE scale is developed in Italy to assess chronic disease patients’ cognitive, emotional, and behavioral engagement. | The 5-item unidimensional tool measures patients’ thoughts about their health status that reflect their cognitive, emotional, and behavioral readiness of health management. It conceptualizes patient engagement as progressing through four experiential stages, i.e., blackout, arousal, adhesion, and eudaimonic project. | The PHE scale can be adapted to assess patients’ readiness to actively engage in managing their health within telecare settings. It can help to evaluate how telemedicine interventions support patients across different engagement stages, from initial confusion to confident use of telemedicine tools. |
| OPTION (observing patient involvement) scale [4] | The OPTION scale is developed to measure the extent to which clinicians involve patients in decision-making during clinical consultations in the primary care settings in UK. | The 12-item unidimensional tool is an observer-rated scale applied to audio/video recordings of primary care consultations. It measures how well clinicians present and explain the health management problem, explore the patient’s expectations and concerns about health management, check the patient’s understanding and foster interaction and joint decision-making. | The OPTION scale can be adapted to evaluate patient engagement from the perspective of observable practitioner behaviors, including their information provision, patient preference elicitation, and parternship, for telemedicine consultations. |
| Perceived Involvement in Care Scale (PICS) [5] | The PICS was developed to measure how patients perceive their involvement during primary care visits in the U.S. | The 13-item instrument has three subscales: doctor facilitation scale, patient information scale, and patient decision-making scale, which measures patients’ perceived involvement in their care management. | The PICS can be adapted to assess patient engagement by evaluating how healthcare practitioners facilitate communication, how patients interact with practitioners, and the extent of shared decision-making in telemedicine settings. |
| Patient Participation Scale (PPS) [6] | The PPS was developed to measure patient participation across hospitalization and ambulatory care in South Korea. | The 21-item scale has four subdimensions: sharing of information and knowledge, performing proactive self-management activities, establishing mutual trust relationships, and partaking in the decision-making process. | The PPS can be adapted in telemedicine research to evaluate patient engagement as a multidimensional construct encompassing information and knowledge sharing, self-management, trust building, and participation in decision-sharing, which primarily emphasizes on the evaluation of patients’ behavioral participation. |

**References**

1. Hibbard JH, Stockard J, Mahoney ER, Tusler M. Development of the Patient Activation Measure (PAM): Conceptualizing and measuring activation in patients and consumers. Health Services Research. 2004;39(4p1):1005–26. doi: 10.1111/j.1475-6773.2004.00269.x.

2. Hibbard JH ME, Stockard J, Tusler M. Development and testing of a short form of the patient activation measure. Health Services Research. 2005;40(6p1):1918–30. doi: 10.1111/j.1475-6773.2005.00438.x.

3. Graffigna G, Barello S, Bonanomi A, E L. Measuring patient engagement: development and psychometric properties of the Patient Health Engagement (PHE) Scale. Frontiers in Psychology. 2015;6:274. doi: 10.3389/fpsyg.2015.00274.

4. Elwyn G, Edwards A, Wensing M, Hood K, Atwell C, Grol R. Shared decision making: Developing the OPTION scale for measuring patient involvement. Quality & Safety in Health Care. 2003;12(2):93–9. doi: 10.1136/qhc.12.2.93.

5. Lerman CE, Brody DS, Caputo GC, Smith DG, Lazaro CG, Wolfson HG. Patients’ perceived involvement in care scale. Journal of General Internal Medicine. 1990 1990/01/01;5(1):29–33. doi: 10.1007/BF02602306.

6. Song M, Kim M. Development and validation of a patient participation scale. Journal of Advanced Nursing. 2023;79(6):2393–403. doi: 10.1111/jan.15593.
